# A Case of Van Wyk-Grumbach Syndrome

**Published:** 2013-06-19

**Authors:** Muhammad Zafar Iqbal, Muhammad Saleem, Zahoor Ahmad Shahzad

**Affiliations:** Department of Pediatric Surgery, Sheikh Zayed Medical College Rahim Yar Khan, Pakistan

**Dear Sir,**

 In boys precocious puberty is defined as the development of secondary sexual characters before 9 year of age. In girls, the development of breasts (thelarche) before 7.5 year, the development of pubic hairs (pubarche) before 8.5 year or menarche before the age of 9.5 year is considered precocious [1]. Hypothyroidism is usually associated with delayed puberty and rarely with precocious puberty [2]. Hypothyroidism with precocious puberty was first described by Kendle in 1905 [3]. Van Wyck and Grumbach reported the association of multi-cystic ovaries with hypothyroidism and precocious puberty [4]. This pathology is more frequently seen in girls. Precocious puberty is always isosexual and characterized by breast enlargement, multicystic ovaries and menstrual bleeding. In boys it may be associated with testicular enlargement and minimal penile enlargement. Herein we report a case of Van Wyk-Grumbach Syndrome.

An 8 year-old-girl with cyclical menstrual bleeding was referred to us from gynecological department for surgical management of bilateral cystic ovaries detected on ultrasound. There were 10cm x 5cm cyst in right ovary and 2cm x1.5cm cyst in the left ovary. The girl was having cyclical monthly menstrual bleeding (up to 4 days) for the last seven months. It was not associated with abdominal pain, vomiting, hematuria or per rectal bleeding. On general physical examination she was a normal looking girl, relatively short statured but well oriented. Her weight was 30 kg (75th percentile) and height 114 cm (less than 5th percentile). Her head circumference was 52 cm and mid arm circumference was 20 cm. Her vitals were normal. She was anemic. On perineal examination, external genitalia were normal. Breast development was at tanner stage III and pubic hair development at tanner stage II. Abdomen was protuberant. Neurological and ophthalmic examinations were normal.

Investigations revealed Hb 10.2 gm %, serum estradiol < 20.00pg/ml, T3 0.260 pgm/ml (range 2-4.4), T4 0.139 ngm/dl (range 0.93-1.7), TSH 785 mIU/ml (range 0.27- 4.2), LH 4.0 mIU/ml, FSH 6.45mIU and prolactin level was 11.2 micro gm/l (range 10.8-12.6). After investigations due to markedly increased TSH level, diagnosis of “TSH induced precocious puberty” was made and oral levothyroxine was started at the dose of 150 ugm/day. Ultrasound of abdomen repeated after 15 days showed multiple cysts in the right ovary with larger one of 8cm x 4cm. Left ovary was normal. The patient was followed on medicine for 6 months. The menarche stopped, height increased and protuberant abdomen became normal. At nine months follow-up, thyroid profile was within normal range. On ultrasound scan the left ovary was normal while on right side there was a small cyst. At last follow up her weight has reduced to 24 kg and height increased up to 124.5cm. She is currently on levothyroxine 50 microgram per day.

Van Wyck and Grumbach proposed an overlap in negative feedback regulation with over production of gonadotropins as well as thyrotropins (both share common alpha subunit) in response to thyroid deficiency [2]. Later it has been observed that GnRH is unresponsive or bionegative and precocious signs are due to thyrotropins. Follicle stimulating hormone theory postulated that high levels of TSH interacts with FSH receptors, inducing FSH like effects on the gonads resulting in multicystic ovaries, menstrual bleeding and breast enlargement. In our patient, TSH was markedly elevated and precocious features were present. Children with precocious puberty due to other etiologies are tall, have pubertal growth spurt and advanced bone age where as children with hypothyroidism are short statured, have delayed bone age and there is no concomitant growth spurt. Treatment of precocious puberty due to hypothyroidism is simply orally levothyroxine.

## Footnotes

**Source of Support:** Nil

**Conflict of Interest:** None declared

